# Rapid Identification of Paragonimiasis Foci by Lay Informants in Lao People's Democratic Republic

**DOI:** 10.1371/journal.pntd.0000521

**Published:** 2009-09-22

**Authors:** Peter Odermatt, Duong Veasna, Wei Zhang, Nanthasane Vannavong, Souraxay Phrommala, Shigehisa Habe, Hubert Barennes, Michel Strobel

**Affiliations:** 1 Department of Public Health and Epidemiology, Swiss Tropical Institute, Basel, Switzerland; 2 Institut de la Francophonie pour la médicine tropicale, Vientiane, Laos; 3 Institut Pasteur du Cambodge, Phnom Penh, Cambodia; 4 National Institute of Public Health, Vientiane, Lao PDR; 5 Department of Microbiology and Immunology, School of Medicine, Fukuoka University, Fukuoka, Japan; Khon Kaen University, Thailand

## Abstract

**Background:**

Paragonimiasis is a food-borne trematodiasis leading to lung disease. Worldwide, an estimated 21 million people are infected. Foci of ongoing transmission remain often unnoticed. We evaluated a simple questionnaire approach using lay-informants at the village level to identify paragonimiasis foci and suspected paragonimiasis cases.

**Methodology/Principal Findings:**

The study was carried out in an endemic area of Lao People's Democratic Republic. Leaders of 49 remote villages in northern Vientiane Province were asked to notify suspected paragonimiasis patients using a four-item questionnaire sent through administrative channels: persons responding positively for having chronic cough (more than 3 weeks) and/or blood in sputum with or without fever. We validated the village leaders' reports in ten representative villages with a door-to-door survey. We examined three sputa of suspected patients for the presence of *Paragonimus* eggs and acid fast bacilli. 91.8% of village leaders participated and notified a total of 220 suspected patients; 76.2% were eventually confirmed; an additional 138 suspected cases were found in the survey. Sensitivity of village leaders' notice for “chronic cough” and “blood in sputum” was 100%; “blood in sputum” alone reached a sensitivity of 85.7%.

**Significance:**

Our approach led to the identification of three previously unknown foci of transmission. A rapid and simple lay-informant questionnaire approach is a promising low-cost community diagnostic tool of paragonimiasis control programs.

## Author Summary

Paragonimiasis is a neglected pulmonary disease provoked by a food-borne trematode parasite. The infection may develop into severe pulmonary disease, often diagnosed with delay and confused with tuberculosis. Globally an estimated 21 millions people are infected. Human infection is acquired through consumption of raw crab, crayfish or wild boar. Typically infections occur clustered in foci of few to several villages where nutritional habits allow transmission. A major challenge for control is to identify the transmission foci. We evaluated a questionnaire approach using lay-informants at the village level to identify paragonimiasis foci and suspected cases. We sent a 4-item questionnaire to 49 village-leaders of a district in rural Lao PDR asking them to report patients with key symptoms of paragonimiasis, i.e. “chronic cough” and “blood in sputum”. The evaluation showed that lay-informants' report had a high sensitivity to identify suspected cases of paragonimiasis using “blood in sputum” as indicator. The approach allowed identifying 3 new, previously unknown foci of transmission in the district. We conclude that lay-informant questionnaires using easily identifiable key symptoms are simple to carry out and are promising low-cost tools for paragonimiasis control.

## Introduction

Paragonimiasis is a food-borne trematodiasis and belongs to the so-called neglected tropical diseases although today an estimated 21 million people are infected [1] and 293 millions are at risk for infection [2]. Six of the 40 known species of *Paragonimus* genus [3] may lead to human infection, and may provoke severe and prolonged lung disease: chronic cough, haemoptysis and protracted pleural effusions. Paragonimiasis is endemic mainly in Asia but transmission foci are also known in African and South-American countries. In endemic areas numbers of cases increased recently such as in India [4], and extra-pulmonary ectopic localizations in skin, liver and brain have been described [5],[6], which may lead to severe disease. Recent studies indicate that full recovery of pulmonary disease can not always be achieved with current treatment options [7].

*Paragonimus* spp. displays a typical trematode life-cycle with two intermediate hosts. Freshwater snails and crabs act as first and second intermediate hosts, respectively. Humans acquire infection by ingesting metacercariae present in flesh of crabs or crayfish. Paratenic hosts such as omnivorous mammals, i.e. wild boars, may transmit the parasite [8]. Dogs and cats are domestic reservoirs.

Due to its symptoms of chronic cough and haemoptysis *Paragonimus* spp. infection is frequently confused with pulmonary tuberculosis and leads to inappropriate treatment [9]. Praziquantel and triclabendazole are efficacious medicines [10].

Lao people possess a deeply culturally rooted habit of raw food consumption including meat, fish, and crabs and crayfish. Transmission of food-borne helminthiasis such as opisthorchiasis [11] or trichinellosis [12],[13] are widespread. Associated with the widely distributed intermediate crab host it can be assumed that paragonimiasis is much more prevalent than currently acknowledged. Diagnosis is rarely made at village and district level where village health workers and health personnel are largely unaware of this lung disease.

We evaluated the performance of a rapid lay-informant questionnaire approach to identify paragonimiasis patients and foci of transmission. A 4-item questionnaire concerning typical paragonimiasis symptoms (chronic cough, haemoptysis, absence of fever) was sent through the administrative system to the village leaders. They were asked to notify patients with corresponding symptoms. In a subsequent step the village leaders' information was validated, and the suspected paragonimiasis patients' sputa examined.

## Methods

### Ethics statement

Oral informed consent was obtained from all study participants enrolled. Ethical clearance for the study and all procedures was obtained from the *Council of Medical Sciences*, Ministry of Health, Vientiane, Lao PDR, including the use of oral consent which was documented on a spreadsheet. Oral consent was used as the illiteracy rate is high in rural Lao PDR. Approval for the study was obtained from provincial and district health authorities and village leaders.

### Study area

The study was carried out between February and April 2005 in Hinheub district (Vientiane province, Lao PDR) with 23,788 inhabitants in 49 villages (National Census Data 2003). It is a rural, mountainous, multiethnic district approximately 120 km north of Vientiane. The district has one district hospital located at the district capital Hinheub Tay.

In 2003 in Naphong village (approximately 20 km southeast Hinheub Tay) 12 paragonimiasis cases were diagnosed in 33 patients suffering from chronic cough [14]. Follow-up investigation showed that four *Paragonimus* species are transmitted in Naphong and two neighbouring villages (*P. heterotremus*, *P. bangkokensis*, *P. harinasutai*, *P. westermani*) by *Potamon lipkei* and *Chulathelphusa brandti* crabs [15] *P. heterotremus* was confirmed in a human infection [16].

### Lay-informant questionnaire to identify suspected paragonimiasis patients

A suspected case of paragonimiasis was defined as a patient with a chronic cough of lasting longer than 3 weeks and/or with a sputum with blood (red brownish colour) without nocturnal fever. The questions of a 4 item questionnaire were: (i) Do you have a cough for more than three weeks? (ii) Do you have bloody sputum? (iii) Is your sputum coloured red or brown? (iv) Do you suffer from nocturnal fever? The questionnaire was translated to Lao. Pre-tests in Vientiane confirmed the clarity of the questions.

The questionnaire was sent from the district health office to the leaders of all villages through the routine administrative system. A cover letter explained the purpose of the questionnaire. Village leaders were requested to ask the 4 questions (with the help of the village health workers) in each household, note any patient answering positively to one of the questions i) to iii) and to send the filled form back to the district health office. The questionnaire was picked up at the office by the research team.

### Validation of village leader's notifications

Villages were ranked according to the number of notified chronic cough patients and grouped in quintiles. Ten villages were identified for the validation: from each quintile 2 randomly selected villages. In each village a medical team (2 medical doctors) carried out a door-to-door survey to identify suspected paragonimiasis patients using the same lay-informant questionnaire. In addition they examined all patients notified by the village leader.

### Laboratory examination

Suspected paragonimiasis cases of the four largest villages underwent a sputum examination. On two consecutive days three sputum samples were collected per patient: first sputum taken on first contact; a sputum container was given to the patient to collect a second sputum sample early mornings; on contact on the consecutive day the third sputum container was collected. Direct examination of (unstained) sputum using a light microscope was performed on site to detect *Paragonimus* ova. Routine examination (Ziehl-Neelsen stain) was eventually performed in the district hospital for each sputum sample to diagnose infection with acid fast bacilli (AFB, *Mycobacterium tuberculosis*). Paragonimiasis was confirmed if at least one *Paragonimus* spp. egg was detected in at least one sputum examination. Pulmonary tuberculosis (TB) was confirmed if at least one sputum sample was positive for AFB. Paragonimiasis cases were treated with praziquantel (3×25 mg/kg/day for 3 days) according to the Lao National Treatment guideline for district hospitals [17]. Patients diagnosed with TB were referred to the TB national program in the nearby provincial hospital of Phonhong.

### Data management and analysis

All data was entered in EpiInfo (version 6.04, CDC Atlanta). All records were cross-checked against original data sheets. Analyses were performed with STATA, version 8 (Stata Corp., College Station, TX, USA). The questionnaire return rate (# returned/# distributed questionnaires), and the questionnaires' sensitivity, specificity, and positive and negative predictive values were calculated. The survey data performed by medical team and results of sputum examination served as gold standard for village leaders' notification and confirmation of suspected paragonimiasis cases, respectively.

## Results

**Figure 1:**
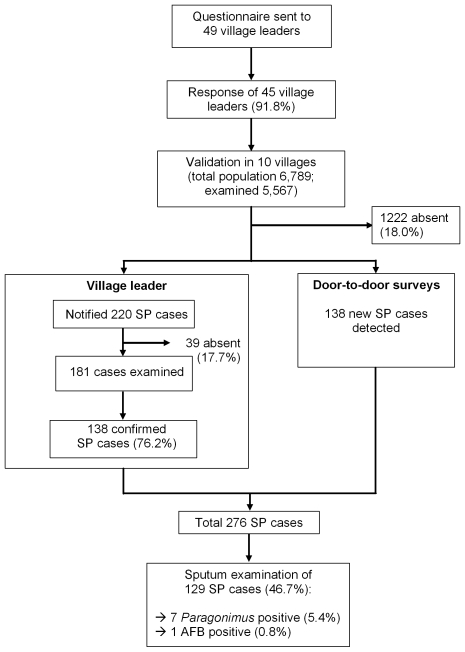
Study flowchart: identification, validation and examination of suspected paragonimiasis cases (SP, suspected paragonimiasis; AFB, acid fast bacilli).

**Figure 2:**
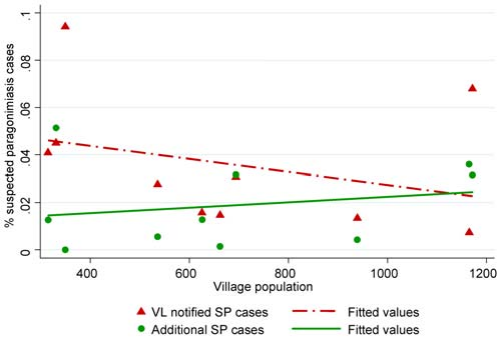
Correlation between village population and portion of notified suspected cases of paragonimiasis (VL, village leader; SP, suspected paragonimiasis).

Figure 1 summarizes the study procedures. 91.8% of village leaders (45 of 49) returned the questionnaire. They notified 220 suspected paragonimiasis cases corresponding to 3.2% of the population of the ten validation villages (range per village: 0.8%–13.8%); 76.2% of whom were confirmed by the research medical team visiting the villages. The number of notified suspected cases of paragonimiasis per population unit decreased with increasing village size (r = −0.329, Figure 2).

Additional 138 suspected paragonimiasis cases were identified during the door-to-door validation survey by the medical team, which were not previously notified by the village leaders. The number of newly identified suspected cases varied between the villages (range: 0–42) and increased with increasing population size (r = 0.72). The number of newly identified suspected cases per population was also positively correlated with village population size (r = 0.21, Figure 2).

In total 276 suspected paragonimiasis cases were found of whom 129 patients sputum (46.7%) examination could be performed (Table 1). Seven patients were *Paragonimus*-egg positive (5.4%) and came from three different villages; 5 of whom were diagnosed in Namthome village (infection rate 10.0%). AFB was diagnosed in one patient from Namthome village.

**Table 1:** Validation and examination of notified and confirmed patients with chronic cough, 10 villages in Hinheub district, Lao PDR.

Village name	Population	Village head	Validation surveys			Sputum examination		
		Cases notified (%)	Confirmed (%)	New cases identified (%)	Total cases	Examined (%)	Paragonimiasis # positive (%)	Tuberculosis # positive (%)
Pakvang	315	13 (4.1)	10 (76.9)	4 (1.3)	14	14 (100)	0	0
Phonethong Tay	331	15 (4.5)	11 (73.3)	17 (5.1)	28	27 (96.4)	1 (3.7)	0
Nampath	349	33 (9.5)	7 (21.2)	0 (0)	7	0 (0)	0	0
Taothane	536	15 (2.8)	10 (66.7)	3 (0.6)	13	4 (30.8)	1 (25.0)	0
Khonekeo	626	10 (1.6)	4 (40.0)	8 (1.3)	12	4 (33.3)	0	0
Houy Yleuth	662	10 (1.5)	7 (70.0)	1 (0.2)	8	5 (62.5)	0	0
Souanmone	694	22 (3.2)	16 72.7)	22 (3.2)	38	19 (50.0)	0	0
Hintid	939	13 (1.4)	9 (69.2)	4 (0.4)	13	3 (23.1)	0	0
Khonekene	1165	9 (0.8)	6 (66.7)	42 (3.6)	48	3 (7.0)	0	0
Namthome	1172	80 (6.8)	58 (72.5)	37 (3.2)	95	50 (52.6)	5 (10.0)	1 (2.0)
**Total**	**6789**	**220 (3.2)**	**138 (62.7)**	**138 (2.0)**	**276**	**129 (46.7)**	**7 (5.4)**	**1 (0.8)**

Table 2 depicts the sensitivity, specificity and predictive values for (confirmed) paragonimiasis of the medical assessment of haemoptysis and the symptoms reported in the village leaders' questionnaire. The analysis was done on all subjects (n = 102) with a complete data set. All *Paragonimus* –egg positive patients suffered from haemoptysis resulting in a 100% sensitivity of haemoptysis assessed by a medical doctor. 34 of 41 suspected paragonimiasis cases with medically assessed haemoptysis were *Paragonimus* – egg negative (positive predictive value 17.1%). Village leaders' diagnostic criteria of “chronic cough” with “blood in sputum” had a 100% sensitivity. Notified cases with “blood in sputum” alone had a lower sensitivity of 85.7%. Village leaders missed to report one paragonimiasis patient suffering from haemoptysis. The utilisation of reports with regard to “fever” did not improve the diagnostic performance of the village leaders. When “no fever” was included as a criteria the sensitivity was lower compared to the analysis where the criteria “with or without fever” was used. All positive predictive values were between 9.7% and 44.4%.

**Table 2:** Diagnostic performance of reported and observed symptoms to identify sputum-positive paragonimiasis patients, Hinheub district, Lao PDR, 2005 (n = 102).

Performed by	Village leader	Village leader	Village leader	Village leader	Medical doctor
Criteria used	Cough + Blood in sputum + without fever	Cough + Blood in sputum with or without fever	Blood in sputum + without fever	Blood in sputum with or without fever	Haemoptysis
**Sensitivity**	71.4 (5/7)	100 (7/7)	57.1 (4/7)	85.7 (6/7)	100 (7/7)
**Specificity**	65.3 (62/95)	31.6 (30/95)	95.8 (91/95)	84.2 (80/95)	64.2 (61/95)
**Positive predictive value**	13.2 (5/38)	9.7 (7/72)	44.4 (4/9)	28.6 (6/21)	17.1 (7/41)
**Negative predictive value**	96.9 (62/64)	100 (30/30)	97.8 (91/93)	98.8 (80/81)	100 (61/61)

## Discussion

In a paragonimiasis endemic district in Lao PDR we evaluated the performance of a rapid questionnaire approach with the objective to identify new paragonimiasis patients and foci of transmission. We used simple questions on “chronic cough”, “blood in sputum” and “absence of fever” used by village leader to screen the village households. Village leaders were highly responsive to our request. A large number of suspected paragonimiasis patients were notified of whom a considerable three-quarter matched our case definition. However, the village leaders missed half of the suspected cases as the door-to-door survey showed. In the sputum examination we confirmed seven paragonimiasis patients in villages where no cases had previously been diagnosed. In one village 5 patients were found. The village leaders' notification of patients with chronic cough and/or blood in sputum had a high sensitivity. The assessment of “blood in sputum” alone resulted in a 100% sensitivity.

Our lay-informant questionnaire approach allowed identifying three new, previously unknown foci of paragonimiasis. In comparison to the high costs of other active case detection methods such as cross-sectional parasitological and/or serological surveys, our approach was performed at very low expenses which were limited to the direct costs of the sputum examination. We conducted the sputum examination in the villages. In a routine implementation of this approach, the diagnosis could be performed at the primary health care level which would further reduce costs. The transport of the questionnaires and screening at village level was performed voluntarily.

Our study benefited from a high response rate of the village leaders. Their notification based on inquiries in the villages with the help of the village health workers was reasonably accurate. Three quarters of the suspected paragonimiasis patients could be confirmed. There was a considerable variation in the number of notified suspected cases of paragonimiasis per population unit. Village leaders from smaller villages reported higher rates of suspected cases indicating that in smaller villagers more efforts could be employed in each household to identify suspected persons. This interpretation is in line with the finding that the medical team found more additional suspected cases of paragonimiasis per population in larger villages. Thus, this questionnaire approach has an optimal functioning in areas with small to medium size villages. Additional activities could be proposed to the village leaders such as village meetings with household representatives to overcome this limitation. In our approach no incentives was given to the village leaders or any activity performed to identify the suspected cases.

It was surprising that village leaders notify only half of the suspected paragonimiasis patients. “Chronic cough of lasting longer than three weeks” and “blood in sputum” seem to be easily recognisable and detectable symptoms. They should be detected also by lay-person. The reasons for this underreporting are not clear but we observed it also in a larger study in six provinces on tuberculosis cases detection using the same question of chronic cough [18]. We suspect that the highly prevalent symptom of chronic cough is very unspecific and often regarded as banal or even “normal”, and therefore not perceived as worthy to notify.

We found that the village leaders report on “blood in sputum” had a higher and in combination of with the presence of “chronic cough” a maximal sensitivity. The inclusion of the presence or absence of reported nocturnal fever did not improve the diagnostic performance. Typically patients suffering from pulmonary paragonimiasis do not have regular fevers [9], in contrast to tuberculosis patients. For this lay-informant case notification process this symptom was not useful.

With this lay-informant questionnaire approach we did not intent to assess the prevalence of paragonimiasis in the community but rather to identify foci of ongoing transmission where later control activities can be instituted, such as information education and communication and/or diagnostic and treatment activities. Therefore, it is a community diagnostic tool applicable for paragonimiasis control.

Similar questionnaire approach has been developed for schistosomiasis haematobium [19]. It is based on the macro haematuria (blood in urine) reported by school-teachers upon interrogation of their pupils. The approach proved to be highly cost-effective. Today, it is applied in the Schistosomiasis Control Initiative in eight African Countries (www.schisto.org). Our approach on paragonimiasis has the additional advantage that it can be combined with tuberculosis. An earlier study showed that the approach increased the detection rate of tuberculosis [18].

Alternatively, an approach using Geographic Information Systems (GIS) in combination with Remote Sensing Techniques (RST) could be developed to delineate areas of transmission of paragonimiasis as it has been done for other trematodiasis. Example given, studies on *Schistosomiasis japonicum* in China showed that the presence of intermediate snail hosts of *Onchomelania* spp. can be predicted by environmental factors such as water availability, vegetation, altitudes, and temperature [20],[21]. A similar mapping tool could be developed for predicting the presence of intermediate snail and crab of *Paragonimus* spp. In combination with information on risky nutritional practices of habit to eat raw foodstuff, accurate risk maps might be developed. The inconveniences of this strategy are the high costs and specialist health professionals involved, both most likely not available locally in Lao PDR and other paragonimiasis endemic areas. In addition, it is not certain that sufficient ecological information on the *Paragonimus* intermediate hosts are available to guarantee the development of such an approach.

Direct sputum examination is known to have a low sensitivity. In particular in children and elderly sputum examinations are often negative or irrelevant due to the fact that good quality sputum are almost impossible to obtain [22]. This fact may explain the relatively large number of *Paragonimus* –egg negative sputum specimen in patient with haemoptysis identified in the village. Thus, additional, more sensitive sputum examination techniques (e.g. coloration, centrifugation, or sputum collection over 24 hours) or serological methods must be carried out [22]. Skin and immunodiagnostic tests based on parasite antigens are highly sensitive [23]. Lately, a molecular diagnostic procedure (PCR) has become available [24].

In the wake of increased awareness for neglected tropical diseases in general and the food-borne trematodiasis in particular, paragonimiasis deserves more attention. The current report shows that the development of a simple community diagnostic tool for improved control of paragonimiasis is feasible.
